# Exploring maternal grief and shame in addiction recovery for pregnant and early parenting women using community-based participatory research: a qualitative descriptive study

**DOI:** 10.3389/fgwh.2025.1565260

**Published:** 2025-09-16

**Authors:** Phyllis Raynor, April Hutto, Khushi Patel, Amber Goforth, Cynthia Corbett, Delia West, Kacey Eichelberger, Constance Guille, Nicole Nidey, Lori Vick, Alain Litwin

**Affiliations:** 1 Center for Advancing Chronic Care Outcomes Through Research & Innovation, College of Nursing, University of South Carolina, Columbia, SC, United States; 2 The Phoenix Center of Greenville, Greenville, SC, United States; 3 Technology Center to Promote Healthy Lifestyles, Arnold School of Public Health, University of South Carolina, Columbia, SC, United States; 4 Prisma Health Upstate, University of South Carolina School of Medicine Greenville, Greenville, SC, United States; 5 Medical University of South Carolina College of Medicine, Charleston, SC, United States; 6 Department of Epidemiology, College of Public Health, University of Iowa, Iowa City, IA, United States

**Keywords:** substance-related disorders, mothers, grief, shame, guilt, mental health recovery, community-based participatory research

## Abstract

**Introduction:**

Little research has explored the constructs of grief and shame-proneness for pregnant and early parenting women seeking recovery from substance use disorders (SUD) and the impact on parents’ and children's well-being.

**Purpose:**

This study aimed to explore the contexts of reported guilt and shame and the associations with grief and loss among pregnant and early parenting women in SUD recovery.

**Methods:**

Using a community based participatory research (CBPR) approach, we conducted in-depth interviews with 30 pregnant and early parenting women with SUD who were recruited from a residential drug recovery facility in the Southeastern United States. A qualitative descriptive six-step thematic analysis established by Braun and Clarke (2006) was used to identify core themes surrounding loss events and feelings of grief, guilt, and shame.

**Results:**

There were 21 (70%) participants that reported at least one significant loss with a total of 56 loss events. Most participants who reported grief associated with a loss also experienced guilt and shame. Events surrounding grief and shame included: losing custody of children, adoption or child death, loss of romantic or familial relationships, guilt from using drugs while pregnant, loss of family support, and perceived loss of maternal attachment. Feelings of guilt and shame were mostly in the context of one's personal feelings of judgement or judgement from their family.

**Discussion:**

Our findings highlight multiple challenges and stigma surrounding maternal SUD, particularly during pregnancy and postpartum. We also attend to the critical need for relevant maternal support to adequately address complicated grief and shame-proneness in SUD treatment to facilitate positive parenting, recovery outcomes, and positive child health.

## 1 Introduction

Women (i.e., assigned female at birth) have the highest risk of developing a substance use disorder (SUD) during their reproductive years (i.e., age 18–44) (1, 2). Approximately 7% of women who gave birth in hospitals had a SUD (1). According to the U.S. Centers of Disease Control and Prevention, the national prevalence of maternal opioid use disorder (OUD) more than quadrupled from 1999 to 2014 (3) and SUD increased significantly between 2018 and 2021 (4).

There are several well-known risk factors associated with substance use in women, including prior unresolved trauma and intimate partner violence (5). Additionally, complicated grief compounded by prolonged feelings of guilt and shame are significant contributors to continued substance use in adulthood. According to Furr et al. (6), complicated grief associated with substance use has been connected to losses that happened early in life or that occurred in adulthood while using substances.

Research on the constructs of guilt and shame-proneness for pregnant and early parenting women seeking recovery from SUD and the impact on parents' and children's well-being remains limited. Developing our understanding of this relationship is critical as overdose remains a leading contributor to maternal mortality. In this study we explored the lived experiences of pregnant and parenting women with SUD using a community based participatory research (CBPR) approach to understand the contexts of reported guilt and shame and the associations with grief and loss. Drawing upon in depth interviews, our findings highlight multiple challenges and stigma surrounding maternal substance use, particularly during pregnancy and postpartum. We also attend to the critical need for relevant maternal support to adequately address complicated grief and shame-proneness in SUD treatment to facilitate positive parenting and recovery outcomes.

## 2 Background & significance

The harmful use of substances can be seen with legal products, such as tobacco and alcohol, and with illicit substances, like opioids and cocaine. Continued harmful substance use despite the negative consequences to the individual and their family constitute medical diagnoses of substance use disorders (SUD) (7). For pregnant and postpartum people aged 35–44, harmful substance use and overdose deaths significantly increased between the years of 2018 and 2021 (4). According to Han et al. (4), most of these pregnancy-associated overdose deaths occurred outside healthcare settings, though often in counties where emergency and obstetric care were available. These findings point to other potential treatment barriers such as stigma, delay in help-seeking behaviors, and socioeconomic factors that may be negatively impacting maternal substance treatment access and retention (4).

One mental health challenge seen commonly in people with SUD is prolonged grief disorder, often called complicated grief (7–9). According to the Diagnostic and Statistical Manuel text revision in 2022 (7), prolonged grief disorder (i.e., complicated grief) occurs when symptoms of grief are protracted, persistent, and cause problems in daily life. Experiencing a significant loss was found to increase the risk of substance use across multiple periods, including pre-SUD, active SUD, and recovery from SUD (6). Denny and Lee (10) suggested that prolonged unresolved grief can lead to the initiation of substance use as a means of coping. A study by Groh and Cunmulaj (5) found that unresolved grief increases women's risk of ongoing substance use (11). Parisi and Sharma (12) found that the link between SUD and grief was bidirectional. People engaging in substance use were at higher risk of developing complicated grief after experiencing loss, particularly if they were using significant quantities of substances prior to the loss event (13). Conversely, complicated grief predicted increases in the harmful use of certain substances, such as nicotine and alcohol (13). Prolonged and unresolved guilt and shame, particularly after grief and loss experiences, have been implicated in the development of mental health problems such as depression (14). Complicated grief and depressive symptoms have been associated with feelings of guilt and shame after a lived experiences of loss (14).

Guilt and shame emotions are often referenced together, but are distinct emotions associated with different attributes and behavioral responses (13). These negative self-conscious emotions result from a self-evaluation of how an experience pertains to oneself in relation to others (15). Shame is often referenced as a negative self-evaluation of oneself (e.g., “there is something wrong with me”) while guilt often refers to a negative self-evaluation of one's behavior (e.g., “there is something wrong with the way I act or behave”) (7, 9, 10). People experiencing guilt-proneness can be more likely to overcome patterns of behaviors that they perceive as negative or hurtful, making amends for past wrongs, whereas people experiencing shame-proneness may continue in dysfunctional behavioral patterns, such as avoiding responsibility for past wrongs and lashing out defensively at others when wrongs are acknowledged (12, 16). It has been argued that both emotions can be either destructive or constructive while in recovery from SUD, depending on how these emotions are processed and managed over time (16).

For this study, we examined complicated grief associated with loss events for pregnant and early parenting women with SUD by exploring the contexts of reported guilt and shame and the reported associations with grief and loss. With a clear relationship between SUD and complicated grief, it is essential to understand the aftermath of guilt- and shame-proneness for pregnant and early parenting women seeking recovery from SUD. For this study, we define “parenting” as lived maternal experiences of women (assigned female at birth) who were currently pregnant or parenting at least one child five years old or younger, and we define the “experience of a loss” as a loss by miscarriage, stillbirth, adoption, the loss of child custody to the state or kinship guardian, or the relational loss or death of a loved one.

## 3 Materials & methods

A community advisory board (CAB) consisting of seven key stakeholders in the SUD recovery community informed the recruitment phase for this project. The functions and purposes of the CAB have been discussed in detail elsewhere (17). We also partnered with pregnant and early parenting women to gain insights into resources needed to support their recovery and parenting in their natural ecosystems (18). Using community-based participatory research approaches to address support needs, including grief and loss resources, in the context of lived experiences in natural ecosystems is critical to promoting positive overall maternal health (18).

The sample size for this qualitative study was 30 pregnant and parenting women seeking recovery from SUD. We recruited from a large OB-GYN clinic in a southeastern state and directly from a residential-based treatment center (in the same area as the clinic) which provides temporary housing support for pregnant and parenting women with SUD and their preschool children. We employed a convenience sampling method at both recruitment sites. A recruitment flyer containing information about the study, eligibility criteria, and contact information was posted at the OB-GYN clinic. Interested individuals could contact the study team by phone or email to learn more about the study. All individuals at the residential treatment center were eligible to participate as the inclusion criteria aligned directly with the treatment center's criteria for temporary residence. Treatment center residents were informed about the study by the center's executive team and instructed that the principal investigator (PI) would be on-site weekly in the conference room at the facility if they wanted to know more about the study. The PI traveled to the residential treatment center to be on-site for recruitment on Fridays based on recommendations from the CAB and the center's executive team (18). Interested individuals came to the conference room to learn more about study participation from the PI. Additional details regarding sampling methods and site recruitment have been published elsewhere (18).

Participants were given an IRB-approved Statement of Research explaining the voluntary nature and purpose of the study and measures to protect confidentiality. After consenting to participate, the lead author (i.e., P.R.) scheduled a time to meet with participants in-person or virtually. The lead author conducted all 30 interviews with participants. Audio data was digitally recorded and professionally transcribed. All audio data collected were for research purposes only, and participants were instructed not to include identifying information. Names and other identifying information, when unintentionally stated by participants during the interview, were removed from the final transcription. Audio data collected were deleted after the verification of final transcriptions. No information was provided to anyone not directly associated with the study without permission from the research participant. Identities were further protected using identification codes instead of participant names during the interview process. All data was identified with an assigned identification code unique to the research participant.

### 3.1 Interview guide

An iterative, semi structured interview guide consisting of open-ended questions was originally developed to gather information on preferred parenting and recovery support from mothers and fathers in long-term recovery (19), and later reviewed and refined by the newly formed community advisory board (CAB) (17). Prior to the current study, the interview questions were re-reviewed by the CAB and community stakeholders, and further refined to include specific questions regarding challenges encountered with attending scheduled postpartum and well-baby visits, and approved as an interview guide for pregnant and parenting participants in recovery from SUD (18). The interview guide consists of exploratory questions about the experiences of parenting while taking care of their children, the greatest challenges with maintaining recovery and parenting children at home, parenting and recovery supports participants desired, and the feasibility and acceptability of receiving these supports through digital technology. The complete interview guide has been published and can be found elsewhere (18).

### 3.2 Data analysis

NVIVO 10 (QSR Int., Burlington, MA) software was used to organize and manage the qualitative interview data for analysis. A qualitative descriptive six-step thematic analysis (20) was used to identify, analyze, and report statements and reflections on loss events, grief, guilt, and shame within the participant interviews (20). The lead author and two graduate research assistants conducted the data analysis. Initial coding was performed line-by-line for each transcribed interview and categorized under the subject headings pre-identified through the interview guide. Data were analyzed separately using recursive thematic analysis and a process of categorization (coding) based on the subject matter and patterns expressed in the texts (20).

Each transcript was reviewed several times, and categories emerged based on common thematic patterns across responses. The concepts of loss, complicated grief, guilt, and shame were frequently reported by participants and were identified and compared across transcripts. Since the high frequency of these concepts was not originally anticipated, we further explored them in the context of the participants' lived experiences. The word and phrase search features were used within the data software platform (i.e., NVivo) to identify specific naming of the constructs by participants (i.e., grief, loss, guilt, shame). We also explored transcriptions to determine if associated words were used to describe these constructs (e.g., shaming, ashamed, embarrassed, broken, failure). Memos were written to analyze the context and meaning and to determine their significance to parenting and recovery capacity. Using descriptive analysis, we explored explicit texts from each interview. Next, we summarized the descriptive findings and interpreted the significance of the patterns and their broader meanings and implications (20).

## 4 Results

Thirty interviews with parenting people with SUD were completed from December 2021 to April 2022. All participants were between 18 and 50 years of age. Most participants identified as being in recovery from SUD for less than six months and were currently living in residential-based supportive housing for parenting women seeking recovery (*n* = 25; 83%). The other five participants reported a minimum of one year of recovery from SUD and were living independently in their communities.

All participants were either pregnant (*n* = 5; 16.7%), perinatal [child < 1 year] (*n* = 13; 43%), or parenting a toddler or a child less than five years old (*n* = 18; 60%) (18). Most participants reported having children across all age categories (*n* = 22; 73%). The participants reported having 1–5 children and many had active cases with the Department of Social Services (DSS). Most of the participants (*n* = 24; 80%) reported losing guardianship of one or more of their older children through kinship adoption or making a voluntary decision to give up their parental rights (e.g., adoption) because they were unable to care for them (18).

Participant #11: “So my 17-year-old and my 12-year-old are with their father. He took custody of them while we were still together, which is why we split up…And then my youngest daughter, she was adopted. So, I don't get to see her which it's hard… I tried to make it an open adoption, but they wouldn't have it. But I know that I gave her the best life possible and that she deserves to have a good life because I couldn't give it to her at that time.”

Participant #6: “I have a 13-month-old daughter, and I have a 12-week-old daughter… my situation is a little bit different, because my children right now are in foster care. Basically, for me in early recovery, like my parenting, I’ve found that I’ve had a new desire and want to be in my children's life, to take care of them, if that makes sense. Spending time planning for them. You could think of it as if I’m still pregnant, a little bit, because my daughter was born three days after I came into treatment.”

Most participants (*n* = 27; 90%) reported harmful use of multiple substances. Most self-reported methamphetamine or cocaine use (*n* = 22; 73%) and opioid use (*n* = 18; 60%), along with the use of other substances including, cannabis (*n* = 8; 26.7%) and alcohol (*n* = 8; 26.7%). Common reported SUD combinations were opiates and methamphetamine, methamphetamine and alcohol, cocaine and alcohol, and opiates and benzodiazepines.

Participant #14: “I was off and on heroin and fentanyl for two and a half years. I smoked weed—I don't know… probably for like 10 years. And before, I was into using heroin and fentanyl, I was on rock… I just got with the wrong person. Before that it was alcohol and pills, mainly alcohol. And that started I guess around 15, and then went until I was 21, almost 22.”

Many participants also endorsed challenges with housing, other social health determinants and prenatal care.

Participant #10: “I was pregnant. And I went through inpatient, and then I went through [redacted] program, but I left too early… So, I went back out…then I was trying to have a baby, and then I was getting high. So, I had a baby crying, and then I'm getting high, and so that really, really was not right. So, I went and lived in a homeless shelter for a while, and then they let me come back here because when I lived in the homeless shelter, I got clean on my own again. So, I was able to come back to the [redacted] program. So, you just have to take—like I was taking baby steps, just one at a time. Yeah. It was like I wasn't only but two months clean, but they let me come back here…This place. This is the primary support. This is my family, my home, everything now. My mom's on drugs. My family's on drugs. I can't go back there. So, the primary source [of support] is this place right here…”

Participant #25: “… I was doing patches at one point in time, and I was doing like three a day. I’d take them and put them on the inside of my gums, and then they absorb into your body that way. And I was doing about a gram to two grams of methamphetamine a day. When I came to terms with the fact that I was pregnant I tapered myself off as much as I could, because I was going to the doctor…And then March comes around and I go into labor. And I had tapered myself off enough that my son was born and he didn't go through any withdrawal symptoms, the shaking and the things that you see that most babies—Because like I said, my intention was to get to the doctor, but I didn't want to go to the doctor with any drugs in my system because I knew if I did my other two children would be gone, the baby that I had was going to be gone. So, I was scared to go get any prenatal care at all. And while I was still thinking I had time, I had time, I had time, I went into labor.”

### 4.1 Losses associated with complicated grief

There were 21 participants (70%) that reported at least one significant loss with a total of 56 reported loss events (see Table 1). The losses were categorized by reported loss type and included death of a child, death of a child's father or close family member, breakup of the relationship with the child's father (who was still in active addiction), child adoption, involuntary child removal from home, child indefinitely living with family member or child's father (i.e., loss of custody to known person), foster care, open DSS case, and abortion. Some child losses reported are listed as unknown due to participants not specifying the type of child loss. There were 4 participants who indicated they experienced a combination of different categories of losses (e.g., adoption and child removal by DSS).

**Table 1:** Losses experienced by pregnant women in substance use disorder treatment.

Loss category reported	Number (*n* = 21)	Notes
Death of child	1	
Death of child's father	1	
Breakup of relationship with child's father	2	
Adoption	6	Participants reported 1 or more (up to 4) child(ren) were adopted
Removal from home	6	Child in Department of Juvenile Justice system; Department of Social Services (DSS) temporarily removed child(ren) from home
Child indefinitely living with family member/father	15	Child(ren) staying with father or participant's parent
Foster care	1	
Open DSS case	1	
Abortion	2	
Combination of any two from different categories	4	
Unknown source of child loss	17	Parents reported loss but did not provide additional information on how the child was lost (e.g., adoption, foster care)
Total	56	

Participants identified child loss or child harm as sources of grief. Child loss or harm included child's death, abortion, adoption, loss of custody, the inability to see the child, losing the “mothering” role for a child lost, lack of maternal attachment, and accidental child exposure to harmful substances. Additionally, other sources of grief included those associated with the break-up or death of a significant other, including a child's father or close family member. These losses were identified as letting go of the child's father (who was still using substances), losing connection with the baby's father, death of child's father, and the death of parent (i.e., stepfather of 20 years).

Participants reported reactions to their grief and loss ranging from “spiraling out of control” to increased drug use, anger, to crying and feelings of shame and guilt (see Table 2).

**Table 2:** Participant-identified contexts and coping resources for grief, loss, shame and guilt.

Domain	Source	Response/Reaction	Coping Resources (used or suggested by participants)
Grief and loss	Loss of child by death When kids were exposed to maternal drug use Perceived loss of emotional connection with child (maternal attachment) Loss of child to adoption Loss of familial and romantic relationships	“wanted to die” “spiraled out of control” Shot up meth Crying “dove into addiction” Shame and guilt Low confidence “I was miserable” “It was a really hard thing to go through, to be pregnant with a child and not be attached to it. It was hard” “And for me that was the hardest thing, letting go of him [child’s father] trying to save him was “drowning myself”	Grief counseling Support groups (e.g., Narcotics Anonymous) Boundary setting (e.g., cutting unhealthy people out of life) Recovery meetings
Shame and guilt	Internal stigma: Not being a “good enough” mother Not having an emotional “attachment” to the child “I still carried that guilt of not raising my son” “I know I’m a failure…” “Forgiving yourself” “My choices” to put self in risky or unhealthy situations “I walked away” from child or children “My confidence is low” “I was afraid someone would judge me”	“I didn’t care” “I wanted to die” “I feel broken” “ashamed” Scared Guilt Depression Anxiety “Putting yourself in a hole crying all day” “It makes my stomach turn” “Shame when you are still using” “you’re so scared”	Interaction with other single mothers in recovery Finding ways to process guilt and shame in healthier ways Support in learning how to live independently and make good decisions on one’s own Medications for mental health disorders and substance use disorders Staying focused on recovery Learning healthy coping strategies to deal with relational conflicts “stressing me” Having a strong accessible recovery network In-home services for the family in recovery (participant and significant other) Positive daily affirmations (saying them aloud and seeing them written) Residential safe place to go for support when you are seeking recovery
	External stigma: Nonparents at recovery meetings For being unable to safely care for child For being unable to stay sober/clean (i.e., in recovery)		
	Provider stigma: For being on medication for an opioid use disorder For being a substance user For using while pregnant

Participant #10: “I had my other two boys… mine didn't get taken away from DSS or nothing. I just kind of walked away because I wasn't—I was getting high… I feel like I wish there were other resources to where we could connect. I could connect with them on a level without having to argue or ask permission with anybody else, from anybody else to talk to my kids. Like he's 13, like yeah, … I know the phone number and I know the address, and so I write letters. But I'm sure that well his mother these days, mom, who has him—and I know that he's not getting my letters. And I know—I don't want to talk to her on the phone because we don't need nobody to argue because then I'm just going to bring that anger up, and it's already there. And then who knows what will happen when I do that.”

Participant #15: “I think it's been great to me [coming to the residential-based recovery support center]. I had several challenges that I didn't even know I had. And I'm happy. Even though at the beginning, I wasn't. I was mad, and I was going through some withdrawals and stuff. Now I see that's it's real life. I'm not numb anymore. That's how I thought about it really… And I used to do it to numb myself because I didn't want to feel the pain. And now my emotions are all crazy because I was a user the whole time since my daughter passed away. I overused my medicine. I just wanted to feel numb. I didn't want to think about it.”

### 4.2 Guilt and shame

Feelings of “guilt” and/or “shame” were reported by 53% (*n* = 16) of participants. Among those 16 participants, 15 (94%) reported at least one “loss” event. Feelings of guilt and shame were in the context of one's own personal feelings of judgement or judgement from their family (*n* = 13; 81%), and judgement from health care providers or peer supports (*n* = 3; 19%). It is important to note that not all participants reported loss event(s) as connected with feelings of guilt and shame. There was one participant who reported “guilt” feelings without connecting it to a particular loss event; this participant experienced loss of a child, but her reported feelings of guilt were associated with using drugs during the first six months of pregnancy.

Participant #3: “I struggled with the guilt of using until I was six months pregnant, so I am trying to get past that…And then one of the things I don't know how to do is how to talk to him [her child] about addiction as he ages. I would love to get some guidance or help about that, especially with his unique circumstances. How do I tell him that I used when I was pregnant, at what age? How do I talk to him about because I used when I was pregnant, you’ve already kind of got that addict programming in your brain”.

Participant #22: “The stress (is the biggest challenge of parenting while in recovery). Like I said, before you could just go get high and relieve the stress. But now we actually have to deal with it, cope with it. But a hard thing, too, in maintaining my recovery and being a parent too is the fact my 7-year-old knows it. So, she does get upset and mad. And then it is kind of like she throws—not that she means to, but I guess this is a natural instinct, but she throws it in my face and then it's like—it kind of breaks my heart because she shouldn't have to go through that. But she did because her mom made bad choices.”

In addition, there was a participant who described shame-proneness although they did not name it as shame. Shame was characterized as being “broken” while addicted or that there was a defect in the person themselves.

Participant #7: “…I know that I wouldn't be this far without people pushin’ me to go forward. Because it's hard to get out of like, when you’re on drugs, you’re broken and you’re low, already. So, somebody has to build you up… For me, … it's a process, … I had to have affirmations on my mirror all the time. I said that I’m beautiful, like, just, it's one day at a time, I take it, and eventually, I built myself back up to where I’m happy in life, and I feel good, and I feel—‘cuz I know before, honestly, the person I was before, I was horrible…you gotta let out and let all that go, and change and remember that I’m not that person. And now I’m happy in life and I feel free. But it's somethin’—it don't just go away. You have to work on it every day. And sometimes, …—your addiction’ll try to drag you back down. You have to keep fightin’…recognition, I’m not that person anymore, and I’ve gotta let go of that; those things that maybe I wasn't so proud of…like, my baby-daddy, had to let it go. Even though I’m takin’ care of the baby on my own, it's better that way, ‘cuz it's better for me to do it alone than to—him to have that brokenness, because his daddy's still broken…And for me that was the hardest thing, letting go of him…I had to realize I can't save them,.[I was] drowning myself”.

Lastly, feelings of regret surfaced in participants when describing parenting while in active addiction vs. being in recovery. Participants characterized feelings of regret in connection to feelings of “being ashamed” for not seeking help for SUD when they felt it was needed and for the low quality of parenting while being in active addiction.

Participant #7: “Even though DSS does so much good for you, at the same time, you’re so scared, you’re lettin’ your child suffer because you’re ashamed to ask for it, because you don't want them to know your business.”

Participant #8: “Your mind, if you’re using [drugs]…you really think that you’re bein’ extra careful, and you’re actually—from my personal experience, know you’re doin’ the greatest job in the world, but you’re not. No, back when—videos of my child when she was little, so much of that—I sit there and I see I’m there. I don't remember a bit of it. Luckily, she was healthy, and I didn't ever harm her. Took care of her, but—I could’ve been more attentive. So, hey, sit there, and play with this, I could’ve been playing with her. But I was too busy gettin’ drunk. So, yeah, that's a big difference. I’m very much more careful. More attentive. My daughter, can't really say I was a bad mother. But I literally put her in her crib and just figure, fine, until she got where she would climb out of her crib up there, and in her play pen. Change her diaper. Never had any problems. Like, I didn't have DSS out here. If she wasn't cryin’, she was fine. I didn't do that extra, hey, let's play kinda deal. Where now, I’ll be like, I’ll be on the floor, playin’ with him…”

### 4.1 Parenting and recovery support to address guilt and shame

Participants described internal (e.g., grief, guilt, shame) and external factors (e.g., stigma, lack of tangible environmental support) that made it difficult to access and receive healthy parenting and recovery support while living in their communities (18). Broad support needs emerged as themes and were categorized as parenting resources, childcare resources, housing supports, recovery supports, occupational training and assistance, and spirituality resources (18). Additional information regarding needed parenting and recovery supports have been published elsewhere (18).

Supports that specifically emerged around the domain of grief and loss included grief counseling, support groups, healthy boundary setting in relationships, and attending recovery meetings to obtain mutual support from other mothers with a shared lived experience with SUD (see Table 2). Supports needed to address guilt and shame included healthy strategies to process guilt and shame without using drugs as a means of coping, receiving medications for mental health disorders and SUD, learning how to live and thrive in one's natural community and make good choices regarding one's health and relationships. Tangible supports described were in-home support services for the family in recovery, and a residential safe place for families (i.e., mothers and fathers/significant others, children) to go for support when seeking recovery from an unhealthy natural environment.

## 5 Discussion

Thematic analysis was used to identify core themes about pregnant and parenting women's context surrounding loss and their feelings of complicated grief, guilt, and shame (21). Most participants who reported a loss and prolonged grief associated with a loss also experienced guilt and shame. Events surrounding grief and shame included: losing custody of children, adoption, or child death, loss of romantic or familial relationships, guilt from using drugs while pregnant, loss of family support, and perceived loss of emotional attachment to their child. Participants identified needed parenting and recovery support to address guilt and shame, particularly grief classes and a supportive recovery network, to help them cope with and process unresolved feelings of guilt and shame as they parented. It is critical that clinicians recognize and respond to complicated grief for pregnant and parenting women, particularly for those who lose custody. Grief acknowledgement, support, and counseling are needed as part of comprehensive SUD treatment (22).

Prior research has addressed the potential impact of the loss of the parenting role on parental efficacy and its negative impact on one's recovery from SUD (23, 24). Ehrmin (23) noted that unresolved feelings of guilt and shame negatively impacted the perceived maternal role and functioning, potentially serving as a significant barrier to successful treatment retention and recovery for a sample of African American parenting women with SUD. Unresolved guilt and shame could also play a negative role in women's perceived ability to parent effectively or in their confidence in parenting, although this is an understudied area of research (19, 24). Because parental efficacy is a predictor of parenting behaviors and subsequent child health outcomes, maternal-focused supportive interventions designed to adequately address guilt- and shame-proneness may improve parental efficacy which offers the potential for overall improvement in family well-being (24). In addition, strategies designed to build positive maternal attachment and bonding in this context have the potential to sustain positive maternal recovery and parenting trajectories and promote healthier early childhood development (25). Further, interventions that promote compassionate self-talk, mindfulness self-compassion, and self-care may go a long way in fostering treatment retention and improving confidence in the ability to parent effectively in recovery (25, 26).

**Figure 1:**
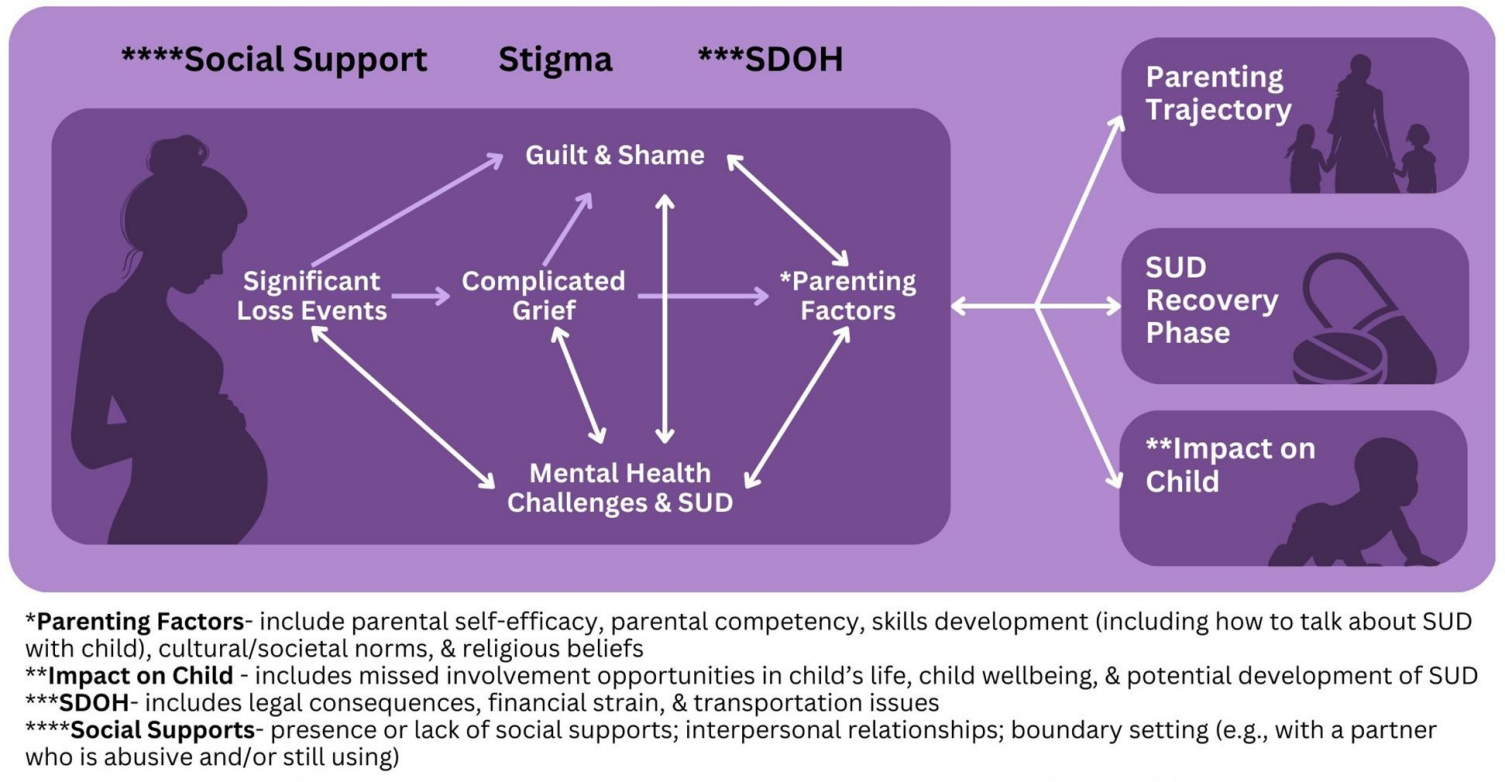
Grief and shame—connections to maternal health factors.

Shame and guilt predominantly revolved around personal feelings of negative judgement (i.e., internal stigma), perceived and explicit judgement from their family (external stigma), and judgement from health care providers (provider stigma) (see Figure 1). This finding is consistent with recent evidence that explored the relationship of internal stigma as it relates to feelings of guilt and shame, particularly for mothers with OUD (21). According to Bakos-Block et al. (21), participants reported that feelings of guilt and shame were present during active addiction and well into recovery. The feelings of guilt and shame served as a major barrier to recovery because it was a trigger to return to harmful drug use as a means of coping. As reported by one of the participants in the current study, it is important to process feelings of guilt and shame as a motivator to stay away from harmful drug use and to frame awareness of one's limitations as a strength.

External stigma refers to the negative attitudes, beliefs and practices that are directed toward a person or group of people by other individuals (27). For these participants, external stigma came from family members, strangers, and health care providers, a finding consistent with other recent studies (27). In the current study, participants reported perceived external stigma from nonparent attendees at drug recovery support meetings when participants addressed parenting related SUD triggers and concerns were discussed. Additionally, participants identified perceived external stigma by other family members who did not have a lived experience of SUD. Lastly, participants reported perceived external stigma by health care providers in situations where the client was taking prescribed medications as treatment for SUD and when they were not taking medications as part of SUD treatment, particularly during pregnancy. As health care providers, it is critical that we utilize trauma informed approaches when engaging parenting women with SUD and eliminate stigmatizing written and expressed language and behaviors to better promote help seeking behaviors and treatment retention (28). The coping resources used or suggested by participants can inform interventions providers could employ within healthcare systems to meaningfully address stigma along with complicated emotions of grief and shame (see Table 2).

While pregnancy can present a strong motivation to access and enter SUD treatment (28), difficult losses and prolonged feelings of grief and shame can increase parental risk of returning to drug use (29, 30). Meaningful supports are needed to help parents stay in recovery. Greater access to mental health support would be particularly beneficial, including medication management, therapy, and SUD treatments that promote the ability to cope effectively with daily life and navigate difficult relationships with significant others, family members, and children of whom they lost custody. Parenting people emphasized the need for strong networks that support their recovery efforts, along with avenues to strengthen relationships with children, whether in their custody or not.

Integrative care approaches are an important and appropriate consideration for providing women with greater access to mental health supports and SUD treatments (31). Integrated clinic models typically have a mental health specialist co-located within primary care clinics to provide mental health support when needed (31). Best practices for integrative care models include co-location of mental health and substance use disorder treatment services in the primary care setting, presence of a licensed mental health clinician, a case management approach to patient care, and ongoing screenings and follow ups with clients for 24 months after consultation (31). In many rural areas, specialty practices are not always readily available, and integrative care models provide a path for women with SUD to get the specialty care services they need in more accessible general care settings (31).

Moreover, our findings highlight opportunities for developing additional grief and emotional support within SUD treatment programs. While we explored the lived experiences of 30 parenting women seeking recovery from SUD, our study included a small, largely homogenous sample of participants from a rural southern state in the United States, with most participants living in a single residential-based recovery support facility and a few residing independently in the community. Participants had to be in current SUD treatment. Exploration of these themes among a more diverse group of parenting women seeking recovery from SUD to determine whether there are additional themes that emerge would be important.

Further research should directly inquire about loss experiences, grief, guilt and shame and how that has shaped women's parental self-efficacy and satisfaction with parenting. The study included a small percentage of participants who self-identified as being from minoritized ethnic and racial groups. Future studies are needed that include perspectives of individuals from more diverse racial and ethnic backgrounds and geographical locations. Further, studies should also examine the longitudinal impacts of grief and shame on parental self-efficacy and SUD recovery among parenting women with SUD. Moreover, the impact of maternal grief and shame on child health outcomes through the SUD recovery phases of the women should be explored. The current study did not distinguish between feelings of guilt and shame; additional research is needed to better understand what factors contribute to each, and how each emotion impacts recovery and parenting, as well as the supports needed to individually address shame and guilt. Future studies should also consider the impact of self-recognition of complex feelings (e.g., shame, guilt, grief) and how emotional intelligence and awareness skills can be leveraged to manage these intense emotions and more effectively process grief.

## 6 Conclusion

Pregnant and early parenting women with SUD face internal, environmental, and social challenges when seeking recovery. It is critical that healthcare providers use a trauma-informed approach when working with this population as many have experienced significant loss, complicated grief, and associated feelings of guilt and shame. Learning to cope with these losses and emotions is an important treatment target in recovery. Strategies that promote compassionate self-talk, mindful self-compassion, and self-care may substantively foster treatment retention and improve women's confidence in their abilities to parent effectively while in recovery from SUD. Recovery initiatives that cultivate emotional wellness and adequately address grief have the potential to enhance resilience, improve mental health outcomes and increase the likelihood of sustained recovery, and positive parenting among women with SUD.

## Data Availability

The raw data supporting the conclusions of this article will be made available by the authors, without undue reservation.
